# Quantifying Seasonal Variation in Insecticide-Treated Net Use among Those with Access

**DOI:** 10.4269/ajtmh.19-0249

**Published:** 2019-07-01

**Authors:** Hannah Koenker, Cameron Taylor, Clara R. Burgert-Brucker, Julie Thwing, Tom Fish, Albert Kilian

**Affiliations:** 1 PMI VectorWorks Project, Johns Hopkins Bloomberg School of Public Health Center for Communication Programs, Baltimore, Maryland;; 2 The Demographic and Health Surveys (DHS) Program, ICF, Rockville, Maryland;; 3 RTI International, Washington, District of Columbia;; 4 Malaria Branch, Centers for Disease Control and Prevention, Atlanta, Georgia;; 5 PMI VectorWorks Project, Tropical Health LLP, Montagut, Spain

## Abstract

Seasonal variation in the proportion of the population using an insecticide-treated net (ITN) is well documented and is widely believed to be dependent on mosquito abundance and heat, driven by rainfall and temperature. However, seasonal variation in ITN use has not been quantified controlling for ITN access. Demographic and Health Survey and Malaria Indicator Survey datasets, their georeferenced data, and public rainfall and climate layers were pooled for 21 countries. Nine rainfall typologies were developed from rainfall patterns in Köppen climate zones. For each typology, the odds of ITN use among individuals with access to an ITN within their households (“ITN use given access”) were estimated for each month of the year, controlling for region, wealth quintile, residence, year, temperature, and malaria parasitemia level. Seasonality of ITN use given access was observed over all nine rainfall typologies and was most pronounced in arid climates and less pronounced where rainfall was relatively constant throughout the year. Peak ITN use occurred 1–3 months after peak rainfall and corresponded with peak malaria incidence and average malaria transmission season. The observed lags between peak rainfall and peak ITN use given access suggest that net use is triggered by mosquito density. In equatorial areas, ITN use is likely to be high year-round, given the presence of mosquitoes and an associated year-round perceived malaria risk. These results can be used to inform behavior change interventions to improve ITN use in specific times of the year and to inform geospatial models of the impact of ITNs on transmission.

## INTRODUCTION

Insecticide-treated nets (ITNs) are the primary tool for malaria prevention in sub-Saharan Africa, with one modeling study showing they account for an estimated 68% of the declines in parasite prevalence seen in the past decade.^1^ However, progress in reducing malaria cases and deaths appears to be stalling,^2^ and it is crucial to identify where gains in intervention effectiveness can be made. Insecticide-treated net use is shaped first by issues of access (people who do not have an ITN available within their household cannot use one); second by priorities for available nets based on age, gender, and family structure;^3,4^ and third by a variety of behavioral determinants, most importantly, perceived risk, perceived mosquito density, and discomfort due to heat.^5^ Assessing determinants of ITN use requires measuring ITN use in the context of ITN access. Recent work has shown that comparisons of ITN ownership (percent of households owning any ITNs) to ITN use (percent of people who used an ITN the previous night) are not helpful programmatically as the two indicators have different denominators, and not all household members may be able to use an ITN even if the household owns at least one.^6^ The primary indicator for quantifying ITN use behaviors is the ratio of ITN use (proportion of the population that used an ITN the previous night) to ITN access (proportion of the population that have access to an ITN within their home, assuming each ITN protects two people). This ratio is regularly above the target of 0.80 in most countries.^7^ However, although it is widely acknowledged that there is seasonal variation in the use of ITNs, it is crucial to isolate the ITN use behavior itself, by comparing use among people who have access to an ITN within their home across different seasons.^8^ In this article, the term “ITN use given access” refers to the proportion of people using an ITN among those with access.

Considerations of seasonal variation on ITN use given access are not new. During the early 1990s ITN trials, Bermejo and Veeken remarked that “malaria is markedly seasonal and has an uneven age distribution, which most trials have taken into consideration; but none of them has taken into account that the use of bed nets and curtains may also be seasonal and age dependent.”^9^ A study in Papua New Guinea observed a drop in overall net use from 75.2% to 79.7% to 46.6% to 67.8% during the hot, dry season.^10^ In response to Bermejo and Veeken, perhaps, studies associated with the early ITN trials demonstrated important links between seasonal variation in vector abundance and perceived malaria risk in Tanzania^11^; in Benin, Ghana, and the Gambia, associations were observed between vector abundance and ITN use,^12–14^ although not always directly related to season. In the mass campaign era, increases in ITN use by children aged less than 5 years were observed in Niger from 15% (first post-campaign dry season) to 55% (first post-campaign rainy season),^15^ from 44% to 53% in Togo,^16^ from 66% to 98% in Burkina Faso,^17^ from 31% to 73% in Benin,^18^ and from 55% to 74% in Zambia.^19^ The implications for survey fieldwork timing have also been noted,^20^ with a mean ITN use:access ratio of 0.91 for Malaria Indicator Surveys (MISs) and 0.78 for Demographic and Health Surveys (DHSs), as DHSs are primarily carried out in the dry season, whereas MISs are fielded in the peak transmission season after the rainy season.^7^ In settings where ITN use was very high, minimal increases in ITN use during the rainy season were observed.^21,22^ MacIntyre noted that seasonal variation in ITN use is pertinent for behavior change efforts—individuals may remain at risk for malaria infection even when nuisance biting is low, contributing to residual transmission.^23,24^ A 2011 meta-analysis found that the main barriers to ITN use when ITNs are available are low perceived mosquito abundance and feelings of discomfort, primarily related to heat.^5^ Qualitative studies have also reported decreased use of available ITNs in the dry season^25–27^ because of several interdependent factors: lower perceived risk, social pressure, hot nighttime temperatures, outdoor sleeping, and movement from outdoors to indoors during the night. Although there is substantial evidence of seasonal variation in ITN use, the extent to which variation is consistent across climatic zones and the magnitude of the variation in different settings, particularly while controlling for background demographic variables (most importantly ITN access), have not been quantified.

This study aims to quantify the seasonal trends in ITN use among those with ITN access (termed hereafter as “ITN use given access”) to determine seasonal patterns in different climate zones, assess correlations with lagged rainfall as a proxy for mosquito abundance, and compare results to observed seasonal malaria transmission patterns.

## METHODS

DHS and MIS datasets and their GPS data were downloaded from www.dhsprogram.com with permission. We included countries in sub-Saharan Africa that had at least two surveys between 2005 and 2017 with GPS data and ITN questions in the household questionnaire. Population ITN use was calculated per the Roll Back Malaria Monitoring and Evaluation Reference Group (MERG) guidance.^28^ Population ITN access was calculated using a slightly revised method. First, consistent with MERG guidelines, the number of ITNs in each household was multiplied by two to obtain a total number of “potential users” in the household. Individuals who had used an ITN the previous night in that household were then assigned “access” equal to one; remaining household members were assigned access equal to one until the number of potential users had been reached, with any additional household members then being assigned access equal to zero, in order of their listing in the household member roster. This creates a subpopulation of individuals within the dataset who, in principle, could have used an ITN, among whom individual ITN use can be assessed, along with household- and community-level covariates. Although no individual level determinants of ITN access should be included in models when using this subpopulation, as it remains impossible in these datasets to determine which unused ITN “spot” belongs to which household member, this revised approach does allow for regression modeling and other analyses using household- and community-level variables. These types of analyses are not possible when using the more widely applied “use:access ratio,” which is simply population ITN use divided by population ITN access. A second advantage is that ITN use among those with access is calculated as a percentage (bounded by 0% and 100%), whereas the ITN use:access ratio often exceeds 1.00, where ITN use behaviors are high and on average more than two people share each ITN.^7^ This revised approach results in population access estimates very close to the standard MERG approach. Example computer code for calculating this indicator and a comparison of the standard and revised indicators are included as Supplemental Files 1 and 2.

The following datasets were downloaded from their respective websites (Table 1):

**Table 1:** Data sources

Dataset	Units	Summary statistic	Source	Website
Monthly rainfall	Millimeter	Mean	Climate Hazards Group	chg.geog.ucsb.edu/data/chirps/
Köppen–Geiger climate classifications	31 climate classifications	Mode	CliMond	www.climond.org
Monthly average temperature	Degrees Celsius	Mean	WorldClim	http://www.worldclim.org/
*Plasmodium falciparum* parasite rate	Percentage of children aged 2–10 years, *Pf* positive	Mean	Malaria Atlas Project	www.map.ox.ac.uk

The rainfall data were downloaded as individual months for each year between 2005 and 2017. The individual months downloaded were averaged into rainfall for each month of the year over one of three time periods for the time frame before the year of survey fieldwork: 2005–2009; 2010–2014; and 2015–2017. Five-year (or 3-year for most recent surveys) averages were used to even out short-term anomalies in the data while accounting for changes in weather patterns and climate.

The rainfall, temperature, and *Plasmodium falciparum* parasite rate datasets were masked to remove large water bodies. The climate classifications were processed without being masked because doing so would have removed large portions of Dakar, Senegal; Dar es Salaam, Tanzania; and Conakry, Guinea.

Then, each of the datasets was extracted at the cluster level. Geographic coordinates for DHS/MIS clusters are displaced randomly to protect respondent confidentiality, within a 2-km buffer for urban clusters and a 10-km buffer for rural clusters. To account for displacement, the mean or mode of each pixel within the buffer was calculated for climate variables. In cases where there were no centroids within the buffer, the value of the pixel at the displacement point was recorded. If this did not yield a result, the value for the point was marked as missing for that dataset. A total of 973 clusters (of 26,294) were missing rainfall, temperature, or climate classification data and were dropped (3.7%).

A latitude categorical variable was created based on Nicholson^29^ divisions for unimodal and bimodal rainfall patterns, with divisions at 9°N, 4°N, 0°N, −4°S, and 14°S to divide the continent into six latitude groups. The Köppen climate classification is a widely used climate classification system, with groupings based on seasonal precipitation and temperature patterns. Monthly average rainfall from the 13 Köppen categories, stratified by the six latitude categories was calculated, graphed, and visually sorted into similar rainfall patterns. From the 44 separate Köppen/latitude graphs, nine typological rainfall patterns were identified. Graphs of the 5-year mean monthly rainfall for the 44 categories and notes on their groupings are available as Supplemental File 3.

Reducing from 44 to 9 rainfall typologies allowed groupings that contained a sufficient number of individuals surveyed in different months of the year across different survey years to permit analysis representing a full 12 months in areas with similar rainfall seasonality. For each of the nine rainfall typologies, the odds of ITN use among individuals with access to an ITN within their households were calculated for each month of the year, controlling for region, wealth quintile, residence (urban or rural), year, and malaria transmission intensity using the Malaria Atlas Project classifications of low, moderate, and high *Plasmodium falciparum* prevalence in children aged 2–10 years. For each rainfall typology, reference categories were set to the month with the lowest adjusted odds ratio to facilitate graphing and interpretation.

To further isolate the effects of month on ITN use given access, separate logistic regression models were run for each rainfall typology. This approach controls for potential bias in estimates for a given month, as averages of ITN use are taken across multiple surveys from different years, and certain months may have smaller amounts of data, or data from only one type of location (e.g., urban centers).

## RESULTS

**Figure 1.**
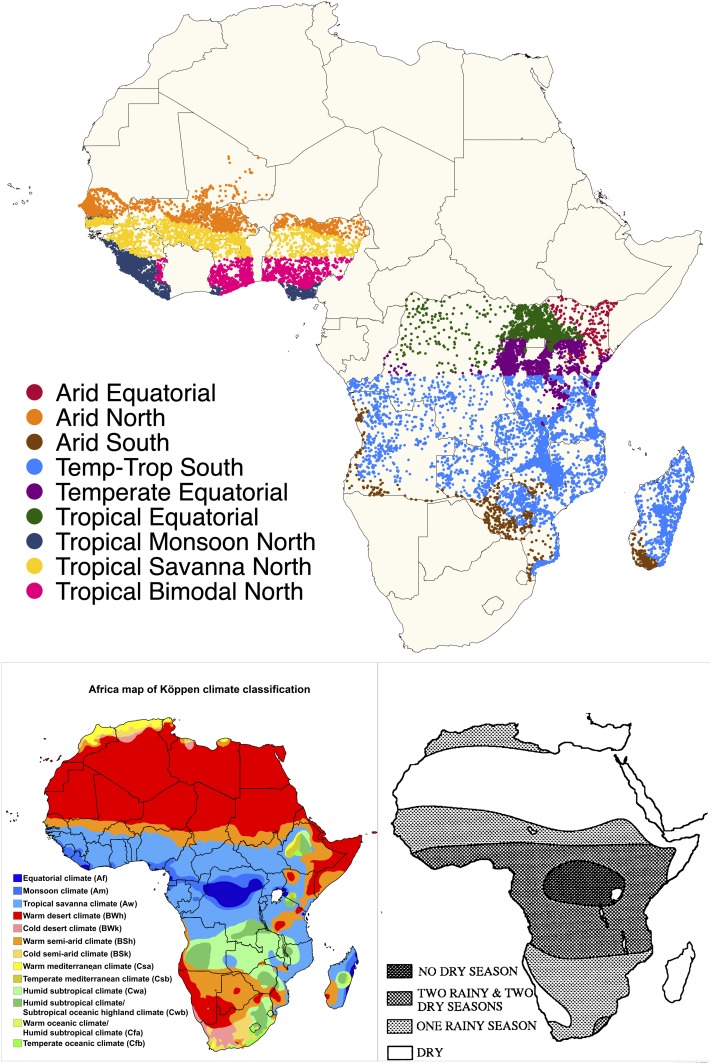
Top: map of included Demographic and Health Surveys and Malaria Indicator Survey clusters (dots) by rainfall typology (colors). Bottom left: Koppen climate classifications for Africa. Bottom right: map of unimodal and bimodal rainfall in Africa, map reproduced from ref. 29, Figure 3. This figure appears in color at www.ajtmh.org.

The total number of individuals in the datasets is presented in Table 2 by rainfall typology and country. Figure 1 maps the clusters according to rainfall typology and reproduces the Köppen and Nicholson maps.

**Table 2:** Percent of surveyed individuals resident in each rainfall typology, by country and overall

	Arid Equatorial	Arid North	Arid South	Temperate–Tropical South	Temperate Equatorial	Tropical Equatorial	Tropical Monsoon North	Tropical Savanna North	Tropical Bimodal North	Total
*N*	32,817	420,053	158,526	1,008,199	533,109	313,577	281,704	399,209	284,692	3,431,886
	Col %	Col %	Col %	Col %	Col %	Col %	Col %	Col %	Col %	Col %
Angola	–	–	27.8	7.9	–	–	–	–	–	3.6
Burkina	–	18.0	–	–	–	–	–	10.2	–	3.4
Burundi	–	–	–	1.1	24.8	–	–	–	–	4.2
Democratic Republic of the Congo	–	–	–	7.3	3.5	13.6	–	–	–	3.9
Ghana	–	–	–	–	–	–	1.4	8.1	25.1	3.1
Guinea	–	–	–	–	–	–	7.8	12.2	1.8	2.2
Kenya	97.3	–	–	0.6	17.8	25.6	–	–	–	6.2
Liberia	–	–	–	–	–	–	35.4	–	2.4	3.1
Madagascar	–	–	21.8	16.7	–	–	–	–	–	5.9
Malawi	–	–	1.5	27.4	–	–	–	–	–	8.1
Mali	–	21.7	–	–	–	–	–	20.0	–	5.0
Mozambique	–	–	7.9	7.6	–	–	–	–	–	2.6
Nigeria	–	19.8	–	–	–	–	18.8	26.4	56.3	11.7
Rwanda	–	–	–	–	26.9	–	–	–	–	4.2
Senegal	–	40.5	–	–	–	–	–	15.2	–	6.7
Sierra Leone	–	–	–	–	–	–	36.6	1.5	–	3.2
Tanzania	2.7	–	–	13.5	20.7	0.5	–	–	–	7.2
Togo	–	–	–	–	–	–	–	6.4	14.5	2.0
Uganda	–	–	–	–	6.1	60.3	–	–	–	6.5
Zambia	–	–	2.7	11.3	–	–	–	–	–	3.4
Zimbabwe	–	–	38.3	6.7	–	–	–	–	–	3.7
Total	100	100	100	100	100	100	100	100	100	100

Col % = column percent

### Insecticide-treated net use given access.

**Figure 2.**
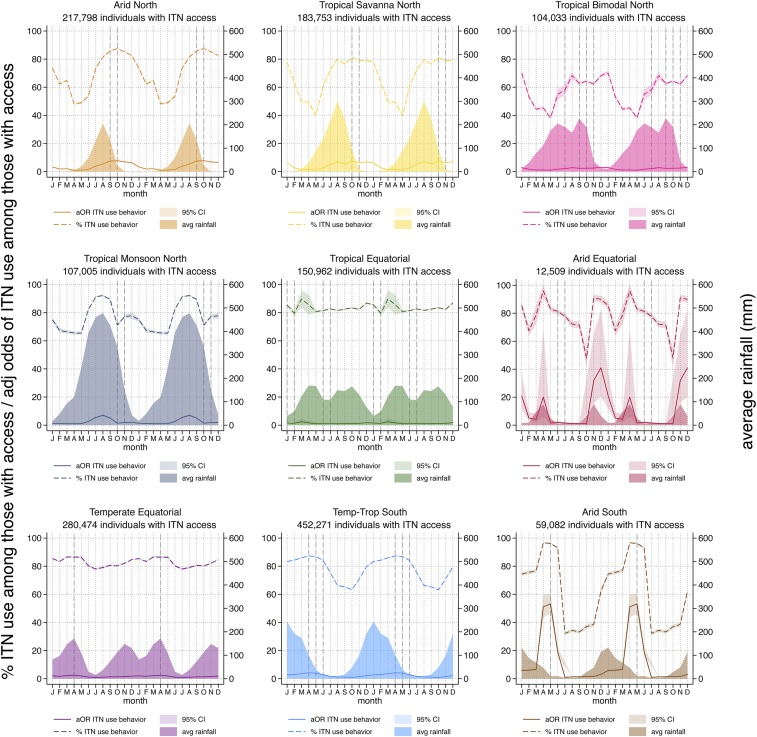
Percent of individuals using an insecticide-treated net (ITN) the previous night among those with access (dotted lines); average rainfall (shaded areas); and adjusted odds of ITN use among those with access (solid lines). Vertical dashed lines indicate the month of peak malaria incidence from selected sites within each zone. To better visualize the relationships between the seasonal patterns, particularly for zones in the southern hemisphere where peak rainfall occurs in December–January, the same 12 months of data are plotted twice in succession. This figure appears in color at www.ajtmh.org.

To isolate seasonal ITN use patterns, the proportion of individuals who used an ITN the previous night among those with access was plotted along with rainfall, shown in Figure 2 (dashed lines). With the exception of the Temperate Equatorial zone, we observe the pronounced dry season declines in the proportion of people with access using ITNs the previous night, with lows around 50% but falling as low as 30% in the Arid South zone. In these same zones, the proportion of individuals using an ITN if they have access is as high as 80% to 100% in the rainy season, except in the Tropical Savanna North, where the maximum is 80%, and Tropical Bimodal North, where the maximum is 70%. In the Tropical Equatorial zone, where the average rainfall is consistently above 40 mm/month throughout the year, very little seasonal variation is observed and ITN use given access remains relatively constant at just above 80%.

### Regression models.

The adjusted odds of individual ITN use among those with access are shown in Figure 2 (solid lines). Trends are similar, with the Tropical Equatorial and Temperate Equatorial zones having the least variation in adjusted odds of ITN use given access, reflecting more or less stable ITN use throughout the year. In the Arid Equatorial and Arid South, the estimates were somewhat unstable, with the adjusted odds of ITN use given access increasing 20–50 fold compared with the dry season. In the Arid North, the odds of ITN use given access were nearly 8 times higher at the peak than the trough and peaked 2 months after the peak rainfall. Seasonality of ITN use given access is also marked in the Temperate–Tropical South, Tropical Savanna, Tropical Bimodal, and Tropical Monsoon, with adjusted odds 4–8 times higher in peak months. Controlling for region, transmission intensity, socioeconomic status, and residence, there are marked seasonal patterns in the odds of ITN use given access.

**Figure 3.**
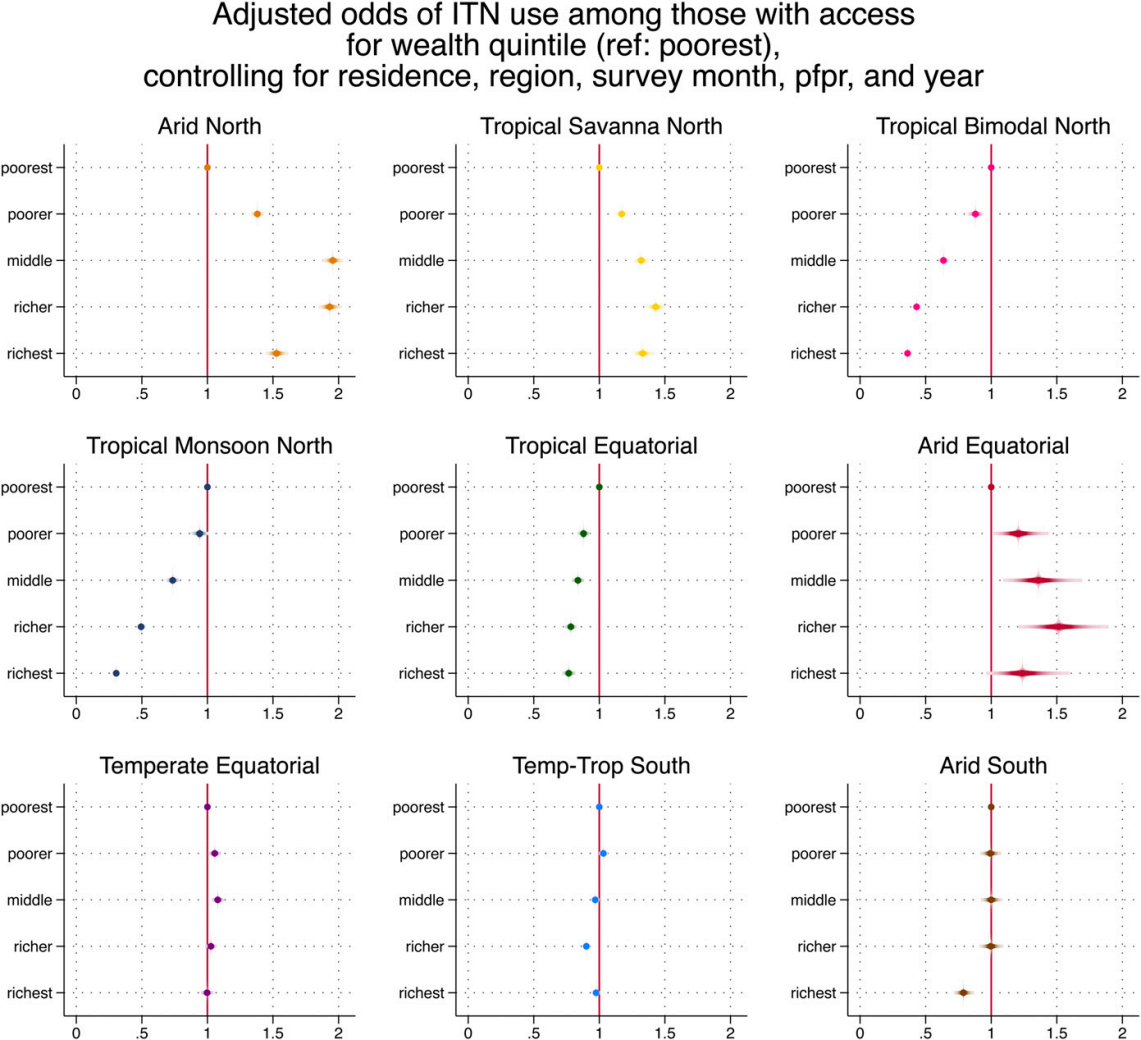
Adjusted odd ratios of insecticide-treated net use among those with access for wealth quintile, by rainfall typology, controlling for residence, region, survey month, *Plasmodium falciparum* parasite rate, and year. This figure appears in color at www.ajtmh.org.

Table 3 presents the full results of the regression models. In eight of nine zones, ITN use given access was lower in rural than in urban areas with the largest difference in the Arid Equatorial zone (aOR 0.38). Regional variation within zones was statistically significant but of a small magnitude. Higher wealth quintiles were negatively associated with ITN use given access in the Tropical Equatorial zone; the association was especially pronounced in Tropical Monsoon North and Tropical Bimodal North (richest quintile aOR 0.26 and 0.378, respectively; Figure 3). By contrast, higher wealth quintile in Arid North and Tropical Savanna North was associated with increased ITN use given access. Compared with clusters of low malaria endemicity, ITN use given access was higher in moderate- and high-risk areas in Temperate–Tropical South and Tropical Monsoon North, but high-risk areas were associated negatively with ITN use given access in Arid Equator, Temperate Equator, and Tropical Savanna North. Insecticide-treated net use among those with access increased over time in all zones except Arid South, Tropical Monsoon North, and Tropical Bimodal North.

**Table 3:** Adjusted odds of insecticide-treated net (ITN) use among those with access to an ITN within their household

Rainfall typology	Arid Equator	Arid North	Arid South	Temperate–Tropical South	Temperate Equator	Tropical Equator	Tropical Monsoon North	Tropical Savanna North	Tropical Bimodal North
Region	**0.93**	**1.00**	**1.02**	**1.03**	**0.99**	**1.02**	**0.95**	**1.03**	**1.04**
Rural residence (ref: urban)	**0.38**	**0.86**	**0.50**	**0.80**	**0.70**	**0.69**	**0.76**	**0.91**	**1.16**
Wealth index (ref: poorest)									
Poorer	1.13	**1.28**	1.01	**1.04**	**1.07**	**0.90**	**0.93**	**1.12**	**0.91**
Middle	**1.35**	**1.74**	**1.14**	0.99	**1.10**	**0.86**	**0.69**	**1.17**	**0.67**
Richer	**1.63**	**1.63**	**1.20**	**0.91**	**1.06**	**0.81**	**0.44**	**1.25**	**0.46**
Richest	1.19	**1.27**	0.92	0.97	1.02	**0.81**	**0.26**	**1.38**	**0.38**
Malaria endemicity*									
Intermediate†	**1.40**	**0.66**	**1.46**	**2.34**	**1.34**	0.97	**1.50**	**0.25**	**0.77**
High‡	**0.32**	1.01	1.05	**2.53**	**0.80**	1.02	**1.44**	**0.33**	0.99
Year of survey	**1.28**	**1.07**	0.99	**1.07**	**1.03**	**1.08**	**0.98**	**1.01**	1.00
Month of interview§									
January	**20.3**	**3.29**	**5.93**	**2.65**	**2.17**	**1.46**	**1.31**	**6.32**	**2.67**
February	**4.88**	**1.75**	**6.07**	**3.05**	**1.90**	**1.29**	**1.18**	**3.03**	**1.64**
March	**3.94**	**2.02**	**6.52**	**3.44**	**2.40**	**2.39**	1.03	**1.35**	**1.12**
April	**19.5**	*ref*	**51.6**	**4.20**	**2.65**	1.67	*ref*	**1.39**	**1.20**
May	**1.67**	**1.05**	**53.9**	**3.98**	**1.93**	*ref*	1.05	*ref*	*ref*
June	**1.98**	**1.57**	**18.7**	**3.03**	**1.15**	1.08	**2.72**	**2.46**	**1.79**
July	**1.60**	**3.49**	*ref*	**1.75**	*ref*	**1.11**	**5.44**	**4.81**	**2.10**
August	1.21	**5.03**	**1.29**	**1.25**	**1.11**	1.05	**7.06**	**6.12**	**2.77**
September	1.08	**7.26**	**1.34**	**1.20**	**1.34**	**1.09**	**5.13**	**5.17**	**2.29**
October	*ref*	**7.73**	**1.55**	*ref*	**1.39**	**1.16**	**1.38**	**7.56**	**2.38**
November	**30.5**	**6.74**	**1.62**	**1.37**	**1.67**	**1.25**	**1.85**	**6.53**	**2.48**
December	**39.3**	**5.59**	**3.08**	**2.18**	**2.01**	**1.73**	**2.00**	**6.84**	**3.00**
Observations	12,509	217,798	59,082	452,271	280,474	150,962	107,005	183,753	104,033

PfPR = *Plasmodium falciparum* parasite prevalence rates among children aged 2–10 years. Bold indicates significance *P* < 0.05.

* ref: low) PfPR_2–10_ ≤ 5%.

† 5% > PfPR_2–10_ ≤ 40%.

‡ PfPR_2–10_ ≥ 40%.

§ ref = lowest month in each zone.

In a separate model assessing rainfall and temperature, but excluding month, increased rainfall (grouped into 50-mm units) was associated with increased odds of ITN use given access in both southern zones, two equatorial zones (Temperate and Tropical), and Arid North (Supplemental File 4a). Rainfall was not associated with ITN use given access in the Arid Equatorial zone and was negatively associated with ITN use in Tropical Monsoon, Tropical Savanna, and Tropical Bimodal. Increased temperature was positively associated with ITN use given access in both southern zones and in Temperate and Tropical Equatorial. The hot season lasts for several months in the southern zones and covers the span of the rainy season; the hot season is shorter in the Northern zones, occurring mainly in the dry season, and ends as the rains begin (Supplemental File 4b). Increased temperature was negatively associated with ITN use given access in the Arid Equatorial and four Northern zones. To assess the interaction between rainfall and temperature, margins were calculated for the interaction term and plotted in Supplemental File 4c. In the Arid Equatorial, Tropical Monsoon, Savanna, and Bimodal, higher temperatures and higher rainfall were associated with lower probability of ITN use, whereas in the Arid South, Temperate–Tropical South, Tropical Equator, and Temperate Equator, lower temperatures and lower rainfall had lower probabilities of ITN use. In Arid North, lower probabilities for ITN use were found at higher temperatures and lower rainfall.

### Countries.

**Figure 4.**
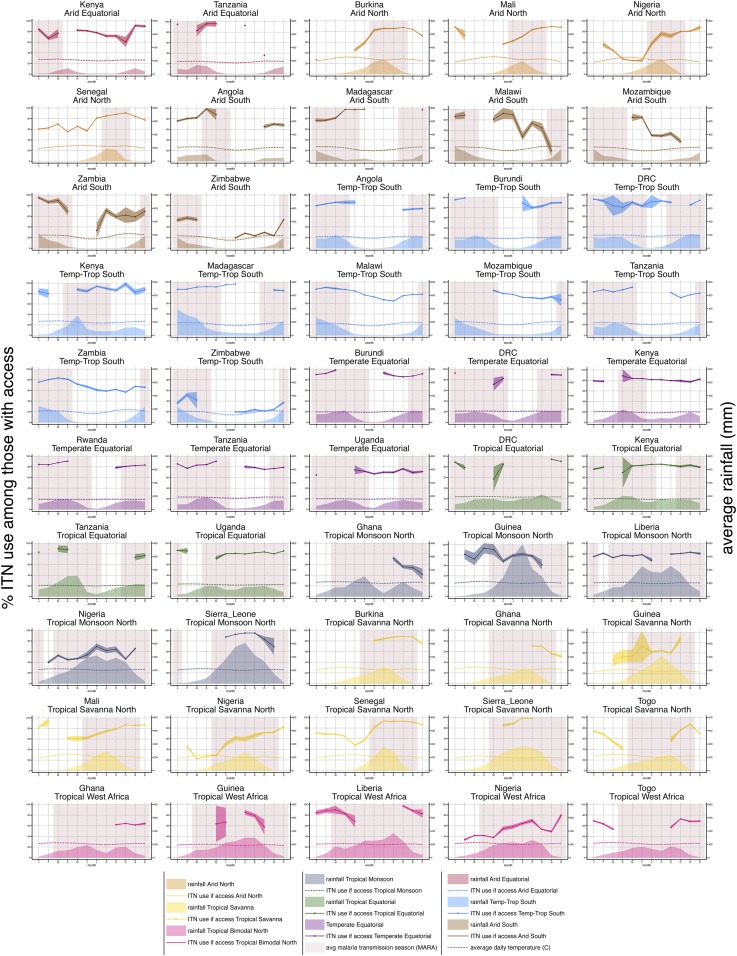
Country-specific graphs plotting mean population insecticide-treated net use among those with access within each climate type (lines with confidence intervals), alongside average rainfall for the same area (shaded areas), mean daily temperature (dotted lines), and average malaria transmission season (red block shading). This figure appears in color at www.ajtmh.org.

Individual country data, pooled from multiple surveys and then stratified by rainfall typologies present in each country, are plotted in Figure 4 against average rainfall and the average malaria transmission season, derived from MARA (Mapping Malaria Risk in Africa) maps of first and last months of transmission.^30,31^ These plots allow for visual inspection of patterns and provide information at a smaller scale for national-level program planners. The lowest months of ITN use given access generally correspond with periods of low malaria transmission. Insecticide-treated net use patterns from different countries with the same rainfall typologies tended to be similar, reflecting the overall pattern highlighted from Figure 2. Sahelian ITN use patterns were consistent between Burkina Faso, Mali, Nigeria, Senegal, and Togo. Angola, Malawi, Zambia, and Zimbabwe had similar curves in their Temperate–Tropical South zones, although the use rate in Zimbabwe was significantly lower than that in other countries. Estimates for Arid South zones across the same countries were somewhat unstable, particularly in Malawi and Zambia, given these zones represented a small relative share of the total population (0.9% in Malawi and 3.7% in Zambia compared with 35.7% in Angola and 47.4% in Zimbabwe). Significant declines in ITN use among those with access to an ITN were observed in certain months in Kenya (August), Malawi (November), Zambia (July), and Rwanda (February). This pattern is due to clustering of location types for that month of fieldwork. In Kenya, clusters interviewed in August were in entirely urban parts of the Rift Valley region. In Zambia, clusters interviewed in July were in entirely rural parts of western province; in Malawi, clusters interviewed in November were in the rural southern region; in Rwanda, February clusters were all in rural Nord province primarily from 2011.

**Figure 5.**
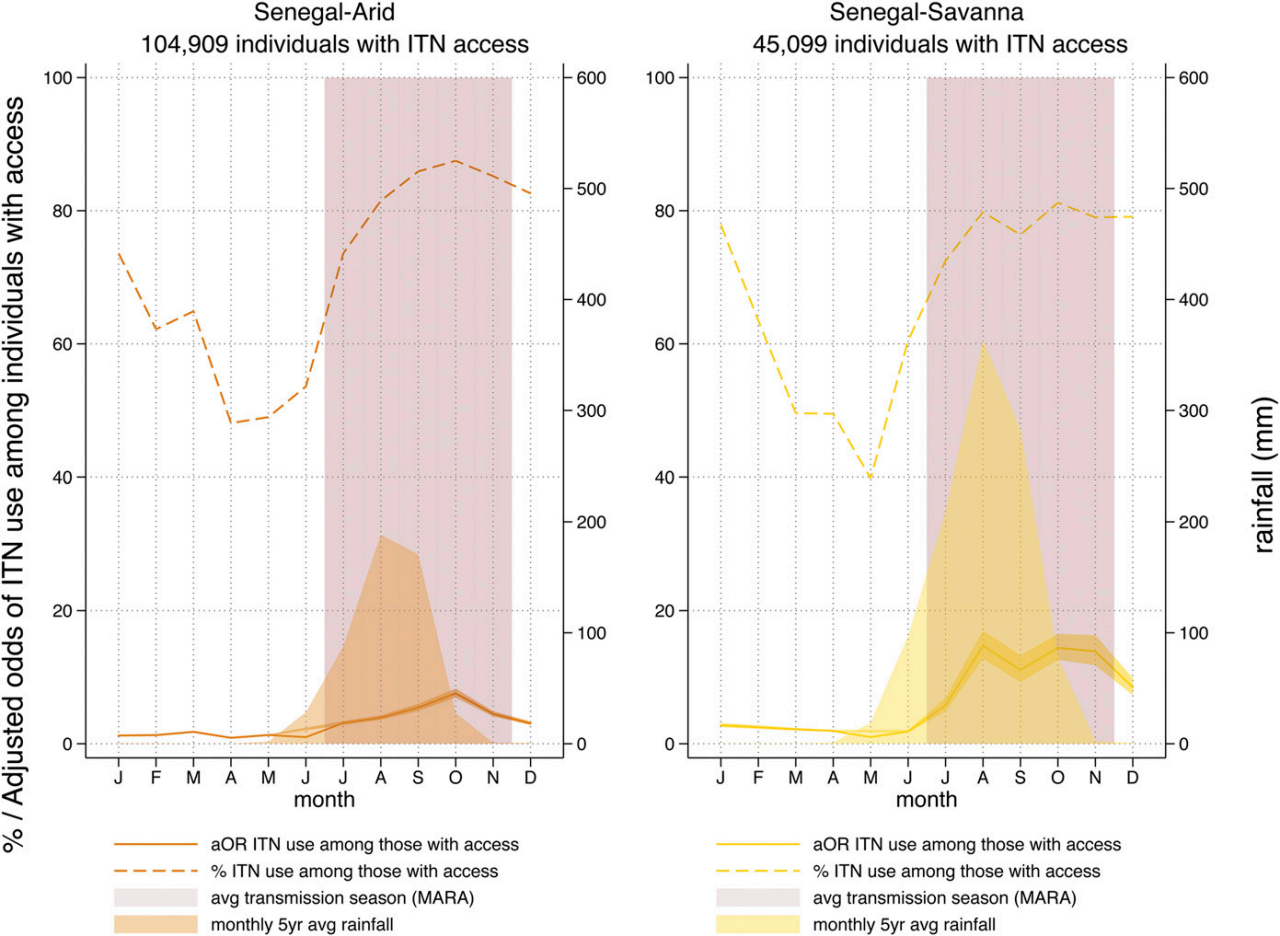
Percent (dashed line) and adjusted odds (solid line) of insecticide-treated net use among those with access in Senegal’s two rainfall typologies. Average rainfall and average transmission season are shown. This figure appears in color at www.ajtmh.org.

Senegal, with seven surveys and the continuous DHSs since 2012, provides the clearest and most complete pattern of ITN use given access. The percentage and adjusted odds of ITN use given access in Senegal are plotted against rainfall in Figure 5 for the country’s two rainfall typologies. The average transmission season derived from the MARA maps is also plotted.^30,31^ Increases in ITN use given access begin just before the first month of malaria transmission and remain high through the final month.

### Lags.

Peak adjusted odds and proportion of ITN use among those with access were similar within each zone. Lags were shortest between peak rainfall and peak ITN given access in the Equatorial and Tropical Monsoon zones, possibly reflecting the fact that mosquito populations are building up more quickly over time in these wet zones than in more arid zones where populations take 4–8 weeks to establish.^32^ The peak malaria incidence for specific sites reported in published literature studies^20,31,33,34^ is included for each rainfall typology in Figure 2. The peak malaria incidence as reported in the literature varied somewhat, given geographic specificities, but tended to align with peak ITN use given access. This may indicate that the perception of risk of malaria is also playing a role in the increased use of ITNs by the population that has access to them.

## DISCUSSION

This is the first study to quantify seasonal variation in ITN use given access, measured as the proportion of the population that used an ITN the previous night among those who had access to one within their household. Seasonal variation in ITN use given access was most pronounced in arid climates. Countries with bimodal rainfall or consistent rainfall had little variation in ITN use given access over the course of the year. Observed lags of 1–3 months between the onset of the rains and increases in ITN use given access are consistent with literature studies on mosquito abundance,^35–37^ suggesting that ITN use is largely triggered by mosquito density. Further supporting this hypothesis are the shorter lags in consistently rainy zones and the low variation in ITN use given access in the zones with the most consistent monthly rainfall. In these areas, ITN use given access is likely to be high throughout the year because of the year-round presence of mosquitoes and an associated year-round perceived malaria risk. Moreover, the positive association of temperature with ITN use in both southern zones and in the larger equatorial zones, where the hot season extends through the rainy season, suggests that people are using nets despite the heat because of mosquito abundance.

Rainfall and temperature influence the breeding and survival of mosquitoes; although excessive rain can wash out breeding sites,^38^ rain creates both humidity and breeding sites necessary for vector reproduction.^35,39–41^ Several climate and meteorological factors, notably rainfall, temperature—particularly minimum temperature—relative humidity, and soil water-holding capacity are linked to the creation and persistence of breeding sites,^42,43^ although the relationship between these variables and malaria incidence is complex.^36,43–45^ It is not surprising that ITN use is linked to rainfall, mediated by mosquito abundance, given the conclusions of Pulford’s meta-analysis that nonuse of ITNs among those with access is driven primarily by perceived mosquito density and discomfort due to heat.^5^ Lagged rainfall has also been associated with malaria incidence, with lags ranging from 1 to 3 months,^34,35,46–49^ reflecting the time for mosquito populations to gradually increase over 4–8 weeks^32,50^ and for parasite development, which also depends on temperature.^51,52^ Recent simple models of vector abundance have used a 1-month lag with climatic variables for both *Anopheles* and *Aedes* mosquitoes.^53,54^

Other studies have documented the motivations for using ITNs to avoid nuisance biting^5,27,55^; net users are unlikely to differentiate between anopheline and other mosquitoes, but the latter are likely to be important drivers of ITN use. There is limited research on the relationship between *Culex* and bednets, despite the importance of the nuisance biter for motivating ITN use,^11^ but one may suppose that rainfall’s influence on *Culex* abundance is unlikely to be substantially different from *Anopheles*. Indeed, the lower adjusted odds of ITN use among rural populations (when wealth quintile is controlled for) may indicate that urban nuisance mosquitoes are a strong driver of ITN use in these areas.

These results clearly demonstrate that ITN use among those with access, although steadily high in some of the highest transmission zones, remains far from target levels—particularly in the drier months of the year—in many countries. The findings may prove helpful in modeling residual transmission potential throughout the year in different environments. Mosquito abundance declines significantly during dry seasons because of lack of rainfall, decreased humidity, and higher temperatures leading to increased evapotranspiration. However, even during the long dry seasons of the Sahel and temperate southern Africa, malaria transmission continues thanks to refugia populations of *Anopheles*, which tend to be found near humans.^56,57^ National programs may wish to use these findings to intensify efforts to improve ITN use in the dry season, or to boost ITN use before transmission peaks and maintain it at high levels through the end of shorter transmission seasons.

The reductions in the odds of ITN use among those with access in higher wealth quintiles in certain zones are hypothesized to reflect reduced perceived malaria risk because of several factors, including improved housing, which has been shown to have a protective effect against malaria,^58,59^ ownership of fans or air conditioners, lower vector densities because of a lack of breeding sites,^60^ and better access to diagnosis and treatment.^61,62^ Several studies have documented lower parasitemia rates in urban zones where most of these highest wealth quintile households live.^63–68^ The two zones in which ITN use is lowest for these quintiles comprise southern Nigeria and southern Ghana, with their highly urbanized centers; recent qualitative research reinforces this hypothesis (Monroe et al., unpublished data). The relationship between wealth and odds of ITN use is reversed in the Arid North and Tropical Savanna, however. We hypothesize that in these drier zones, the poorest households may be largely living in the driest and hottest climates, where mosquitoes are relatively fewer, and are therefore less likely to be using ITNs than wealthier households in the urban areas of interior west Africa.

Controlling for other factors, we observed an increase in ITN use given access in more recent years in the Arid Equator, Arid North, Temperate–Tropical South, Temperate Equatorial, Tropical Equatorial, and Tropical Savanna zones. This may reflect slight improvements in ITN use given access over time, potentially due to ongoing behavior change activities, or by strengthening social norms, particularly as ITN access grew. However, the results on the whole may indicate that ITN use patterns are relatively entrenched.

The most important implications of this work are 2-fold. First, these findings can be used to inform the timing and content of social and behavior change interventions to improve ITN use during drier months of the year. Programs should consider that it is not cost-effective to promote ITN use in locations and in months when it is already high. In order for behavior change interventions to work, there must be “room to move” the population on the target behavior.^69^ There is little “room to move” in the rainy seasons and high transmission seasons, as ITN use given access ranges from 80% to 100% in these months. There is, however, considerable room for improvement in maintaining high levels of population ITN access, which reach only to 80% after mass campaigns and decline thereafter because of net wear and tear and attrition.^2,8^

More work is needed to correlate these ITN use patterns with models of seasonal malaria incidence. It would be critical, for example, to boost ITN use among those with access during times when transmission is beginning to increase but ITN use is not yet widespread. This may mean that programs may need to redirect behavior change efforts toward the early rainy season, as mosquito abundance and transmission ramp up, and work to convince populations that it is worthwhile to use ITNs even when mosquito abundance and perceived risk may be quite low. Residence and wealth quintile were significant determinants in the multivariate models, and programs should look to identify specific subpopulations where ITN use given access is lower for additional behavior change interventions, and work to maintain strong behaviors in populations that are using their ITNs well. Likewise, programs must take into account the season in which data collection is carried out—and especially the timing of fieldwork across different areas of the country—when interpreting data on ITN use among those with access.

Second, these findings may inform modeling of the impact of ITNs on transmission and residual transmission models. Models of malaria intervention effectiveness should take into account the fact that nets may not be used all year round. Models could likewise estimate the marginal impacts on transmission by improving ITN use given access in the dry season, “shoulder” season, and rainy season, and the cost-effectiveness of such efforts. It would be important for any models to take into account the heterogeneity of transmission in a given area.

### Limitations.

There are several limitations to this study. First, the geographical groupings are broad and there is significant heterogeneity within each zone, but the goal was to conduct a broad characterization, not attempt to determine individual-level factors related to rainfall. Second, using pooled data from the available surveys results in overrepresentation of certain countries in certain rainfall typologies, for example, Senegal with its six surveys, or Kenya comprising the Arid Equatorial rainfall typology. There is possible bias in this approach to analyzing ITN use given access if those behaviors are markedly different in one country versus another. We have attempted to address this with the country-specific analysis, where there is no significant variation in seasonal patterns between countries. Although weights were not deemed crucial given power provided by the number of observations, it is possible that weighting by representation in the rainfall typology may shift the overall results. Third, variation in cluster composition over different survey months, even when pooled, may contribute to bias in the proportion of individuals using an ITN among those with access. We have addressed this with the multivariate regression to control for these background factors. It is further possible that unmeasured factors, such as knowledge, attitudes, exposure to social behavior change interventions, and altitude, are also at work. Subsequent studies should further investigate the extent to which these factors are important determinants of ITN use given access. No random or fixed effects were included at household or cluster level, although it is logical that these would be important. Random effects at the cluster level did not substantially change the seasonal use ITN trends (Supplemental File 4d).

Fourth, rainfall averages used in this analysis may hide interannual variation in rainfall that may influence ITN use given access on a more micro level. However, this article attempted to characterize the broad seasonal patterns of different climate zones, making the 5-year average an appropriate instrument, minimizing noise. Subsequent studies where monthly ITN use and access data are available may wish to examine year-to-year variation induced by changing rainfall patterns.

Finally, the limitation inherent in the calculation of ITN access, where no individual-level analyses can be conducted on DHS/MIS data as “access” cannot be known empirically for different household members, means that it is not possible to assess whether seasonal patterns differ by age group or gender. However, given recent studies documenting lower ITN use among children of school age across sub-Saharan Africa,^3^ and their importance as a reservoir of transmission,^70^ future studies may wish to identify methodologies to examine this more closely.

## CONCLUSION

Controlling for region, transmission intensity, socioeconomic status, and residence, there are marked seasonal patterns in the odds of ITN use among those with access to an ITN. In the Tropical Equatorial and Temperate Equator zones where rainfall is relatively constant, seasonal variation in ITN use given access were minimal. Peak ITN use among those with access occurred 1–3 months after peak rainfall, suggesting a link with mosquito abundance and potentially perceived malaria risk. These results should be used to inform social and behavior change interventions to improve ITN use in specific times of the year, and to inform geospatial models of the impact of ITNs on transmission, nationally and globally.

## Supplemental materials

Supplemental materials
