# Supplementary material for: Quantifying Seasonal Variation in Insecticide-Treated Net Use among Those with Access
Source: Am J Trop Med Hyg. 2019 Jul 1;101(2):371–82. doi: 10.4269/ajtmh.19-0249 (PMC6685578; doi:10.4269/ajtmh.19-0249)

The following are supplemental materials and will be published online only

Supplemental File 1

Stata do-file for population ITN access calculation

```
*** version 11
*** Author: Albert Kilian and Hannah Koenker
*** Date: Feb 27 2016

*** DO FILE FOR CALCULATING A REVISED access indicator that takes into account who is
actually using an ITN
*** Ensure you have the following variables already created
	* variable "nitn" is the sum total of ITN in each household from the household
file
	* variable "stay" is the de-facto residency (slept here last night) yes=1, no=0
	* variable "hhid" is the unique identifier for the household
	* variable "persid" is the unique identifier for the individual
	* variable "useitn" = hml19

	gen line=hvidx

	save, replace
*** LOAD the household member (PR) file


*	gen potuse=nitn*2
*	label var potuse "potential ITN users in hh"

*	save, replace

***  discard household members that weren't there; we only calculate this indicator
among de jure members, those who were there the previous night
	keep if stay==1
*** access is 1 if _n (number of hhid records, i.e. people who stayed in the household
last night) is less than or equal to potuse, and 0 if not.
	bysort hhid: gen access=_n<=potuse
*** for each member. So the last listed persons are not going to have access.
*** It goes from the first to last line number within households and allocates 1 until
"potuse" is reached or there are no more people
	label var access "has access to ITN"


	keep access hhid line
	save access, replace

********
******* CLEAR and LOAD household member file again
********

	merge 1:1 hhid line using access
	drop _merge

	save, replace

*** create the adjusted access indicator
*** First calculate number of itn users in each household

	egen nitnuse=sum(useitn), by(hhid)
	label var nitnuse "number of itn users in hh"
*** gen potuse=nitn*2
*** (if you did not run first the old version, this line is needed to calculate
potuse2)
	** subtract the number of itnusers from the number of potential sleeping spaces
under a net to get the remaining nonusers
	gen potuse2=potuse-nitnuse
```

```
      ** if more people used nets than there are potential sleeping spaces under nets,
there are no people who are "potential ITN users who didn't use a net"
      replace potuse2=0 if potuse2<0
      label var potuse2 "potential ITN users in hh who did not use ITN"

*** drop those who used itn or did not stay in house
      save, replace
      keep if stay==1
      keep if useitn==0
*** now we have only people who were there, but didn't use an ITN. we will next assign
them access to the remaining net spaces.
*** for the people remaining, if their count is less than or equal to the number of
people who could've, but didn't use a net, then they get assigned access.
*** we call this access3 because access2 was the official MERG version of calculating
the non-adjusted access
      bysort hhid: gen access3=_n<=potuse2

      keep access3 hhid line
      save access3, replace

********
******* CLEAR and LOAD household member file again
********
            * merge the access3 small file back into the indiv file, matching on hhid
and line number

                  merge 1:1 hhid line using access3
                  drop _merge

                  label var access3 "has access to ITN"
            * assign access to all people who used an itn last night // we have
calculated access for those who didn't use a net, and next:
            * we assign access=1 for those who did use a net.
                  replace access3=1 if useitn==1 & stay==1

                  save, replace


*******
*** ANALYSIS
*******
            *svy: mean access2 if stay==1
            *** compare the original access to the new access
                  svy: mean access2 if stay==1
                  svy: tab access3, perc ci
            *** The subpop is needed because useitn includes those not staying
                  svy: tab useitn, perc ci subpop(stay)
            *** now there can't be anybody who used an ITN and did not have access
and because access3 is only calculated for those who stayed, the stay argument can be
omitted
            *** IMPORTANT - should not be used to determine any individual-level
factors for access (or use among those with access)

                  svy: tab useitn, perc ci subpop(access3)
*************
* end do file
*************
```

Supplemental File 2

Comparison of ITN use:access ratio and ITN use among those with access (adjusted)

In the chart below, the values for ITN use given access (the proportion of people using an ITN the previous night among those with access to an ITN within their household) are plotted against the ratio of population ITN use to population ITN access, from 98 large household surveys (MIS and DHS). In the latter, population ITN access is calculated according to RBM MERG guidance. In the former (y axis), population ITN access is calculated as described in this paper and the do-file in Additional File 1, whereby defacto household members that used an ITN are assigned access=1, and remaining "ITN spaces" (based on number of defacto household members and number of ITNs in the household) are then 'filled up' with individuals in order of their listing in the household roster.

The green circles are the survey results. The pink dashed line is the quadratic function. The gray solid line is the linear function. The quadratic and linear functions are shown in the chart title area. The two indicators are highly correlated, with a slightly better fit for the quadratic function as compared to the linear function (R^2=98.0% for the quadratic function vs 96.1% for the linear).

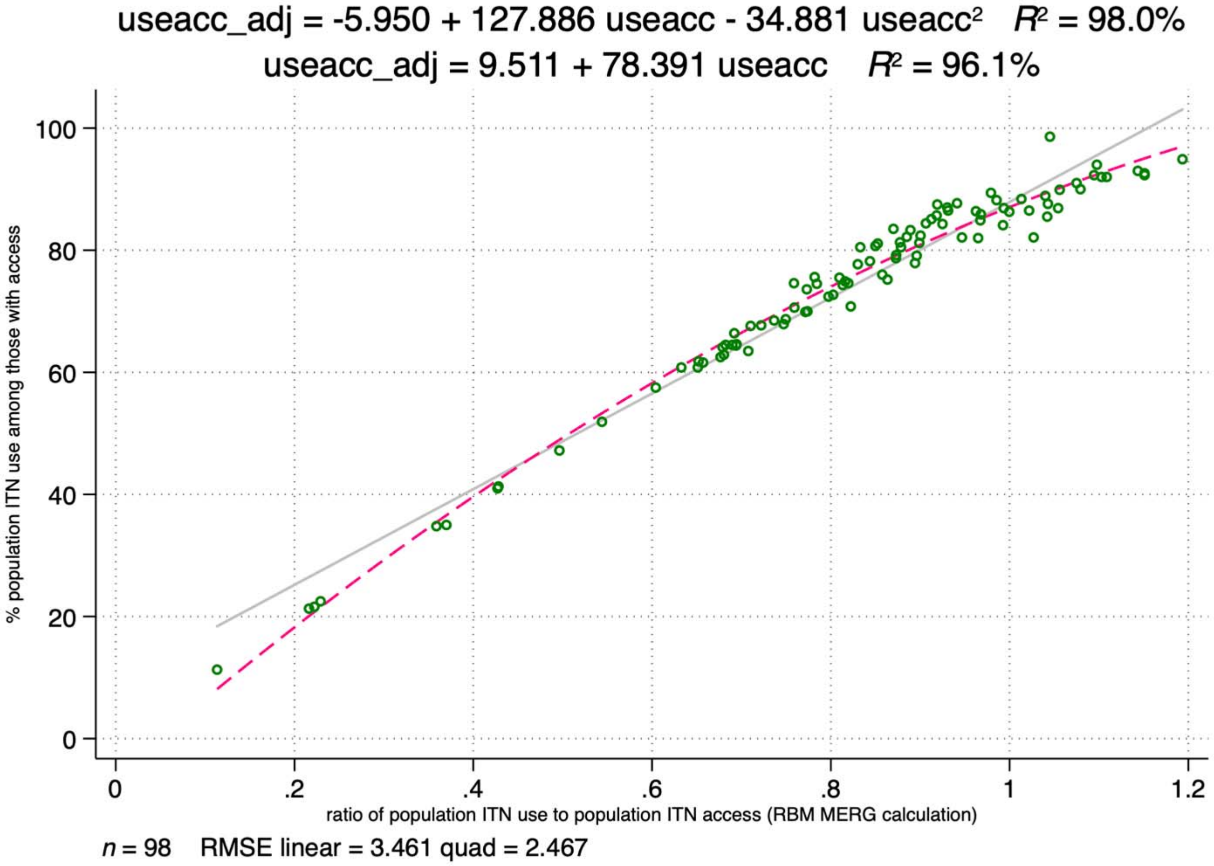
useacc_adj = -5.950 + 127.886 useacc - 34.881 useacc² R² = 98.0%
useacc_adj = 9.511 + 78.391 useacc R² = 96.1%
% population ITN use among those with access
0
20
40
60
80
100
0
.2
.4
.6
.8
1
1.2
ratio of population ITN use to population ITN access (RBM MERG calculation)
n = 98 RMSE linear = 3.461 quad = 2.467

Supplemental File 3

Koppen climate classification rainfall graphs (13 climate categories x 6 latitude categories) with color-codes for reducing to 9 rainfall typologies

In this series of figures mean monthly rainfall is plotted, stratified by 13 Koppen climate zones x 6 latitude categories, to obtain rainfall patterns for different climates in northern, equatorial, and southern zones. The colored dots indicate where rainfall patterns were similar and were matched to create the final nine rainfall typologies. The colors of the dots correspond with those in Figure 1. The Eastern coast of Madagascar (turquoise dots with pink edge) have significantly more rainfall than other Temp-Trop (turquoise) areas. The authors would have preferred to analyze this coast as a separate group; however, the group was missing DHS/MIS data for three months out of the year. For this reason, it is included as part of the Temp-Trop South grouping.

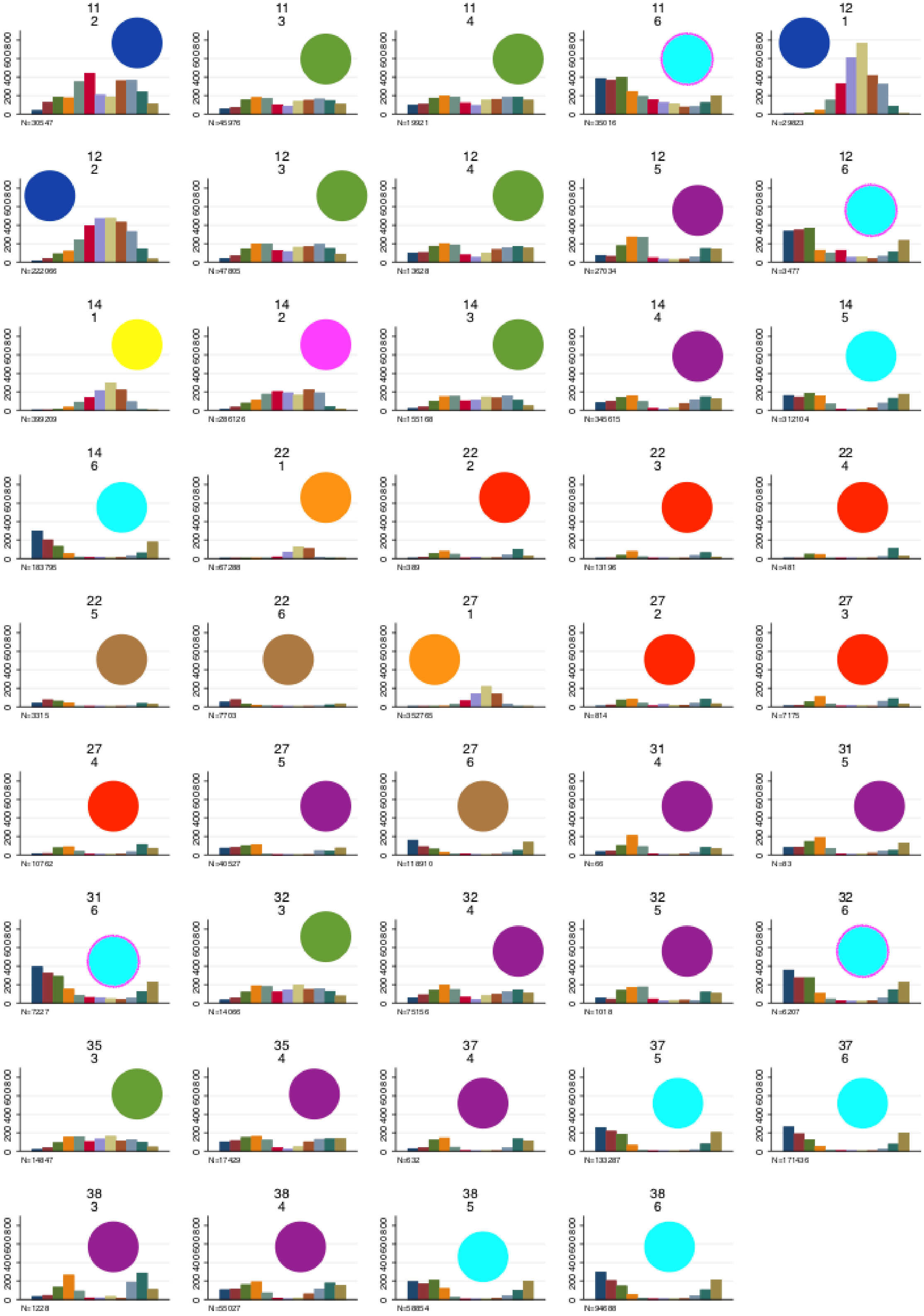

11 2
11 3
11 4
11 6
12 1
12 2
12 3
12 4
12 5
12 6
14 1
14 2
14 3
14 4
14 5
14 6
22 1
22 2
22 3
22 4
22 5
22 6
27 1
27 2
27 3
27 4
27 5
27 6
31 4
31 5
31 6
32 3
32 4
32 5
32 6
35 3
35 4
37 4
37 5
37 6
38 3
38 4
38 5
38 6

SUPPLEMENTAL FILE 4

A. Regression model including temperature, rainfall, and temperature*rainfall, but not month.

Rainfall Typology	Arid Equator	Arid North	Arid South	Temp-Trop South	Temperate Equator	Tropical Equator	Tropical Monsoon North	Tropical Savanna North	Tropical Bimodal North
Region	**0.98**	**1.04**	**1.05**	**1.03**	**0.98**	**1.02**	**0.87**	**1.06**	**1.01**
Rural residence (ref: urban)	**0.34**	**0.89**	**0.56**	**0.85**	**0.71**	**0.69**	**0.85**	**0.93**	**1.18**
Wealth Index (ref: poorest)	1.12	**1.32**	0.96	**1.03**	**1.10**	**0.95**	0.98	1.03	**0.85**
Poorer	**1.22**	**1.89**	0.96	**0.98**	**1.13**	**0.94**	**0.79**	**1.12**	**0.60**
Middle	**1.39**	**1.86**	1.00	**0.93**	**1.08**	**0.89**	**0.55**	**1.31**	**0.40**
Richer	1.12	**1.41**	**0.90**	**1.04**	**1.05**	**0.86**	**0.34**	**1.53**	**0.34**
Richest	**1.14**	**1.06**	**1.06**	**2.02**	**1.12**	**0.80**	1.10	**0.52**	**0.59**
Malaria Endemicity[a]									
Intermediate[b]	**0.34**	**1.94**	**0.77**	**2.18**	**0.79**	**0.79**	1.05	**0.85**	**0.75**
High	**0.89**	**1.09**	**0.98**	**1.04**	**1.02**	**1.07**	**0.96**	**1.07**	**1.01**
Average rainfall in groups of 50mm	0.87	**3.26**	**10.7**	**1.22**	**1.38**	**1.31**	**0.76**	**0.16**	**0.46**
Average temperature (degrees C)	**0.94**	**0.88**	**1.17**	**1.04**	**1.07**	**1.13**	**0.65**	**0.71**	**0.77**
Average rainfall (50mm) x average temperature	1.01	**0.96**	**0.91**	**1.00**	**0.99**	**0.99**	**1.01**	**1.07**	**1.03**
Observations	12,509	217,798	59,082	452,271	280,474	150,962	107,005	183,753	104,033

B. Average monthly temperature and average monthly rainfall in the nine zones

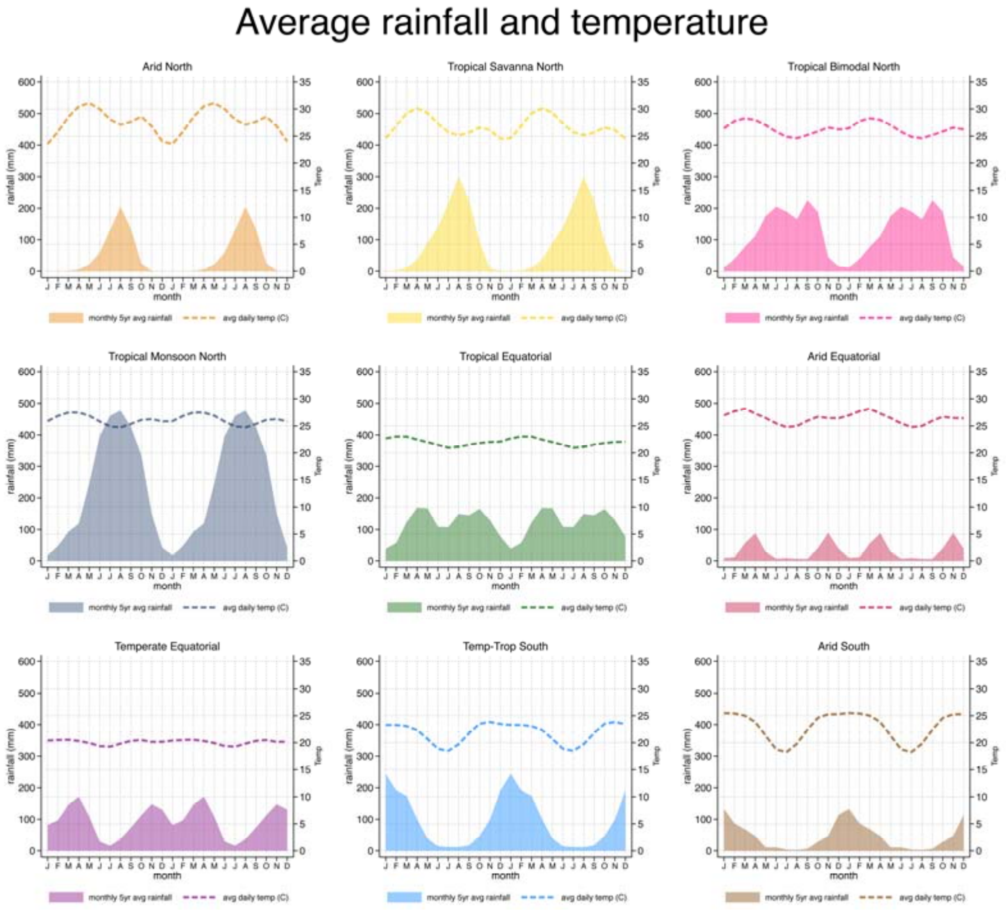

C. Contour plots of margins for rainfall and temperature from the logistic regression models. The effect of temperature on the probability of ITN use given access differs at different levels of rainfall, and these associations are markedly different between different zones.

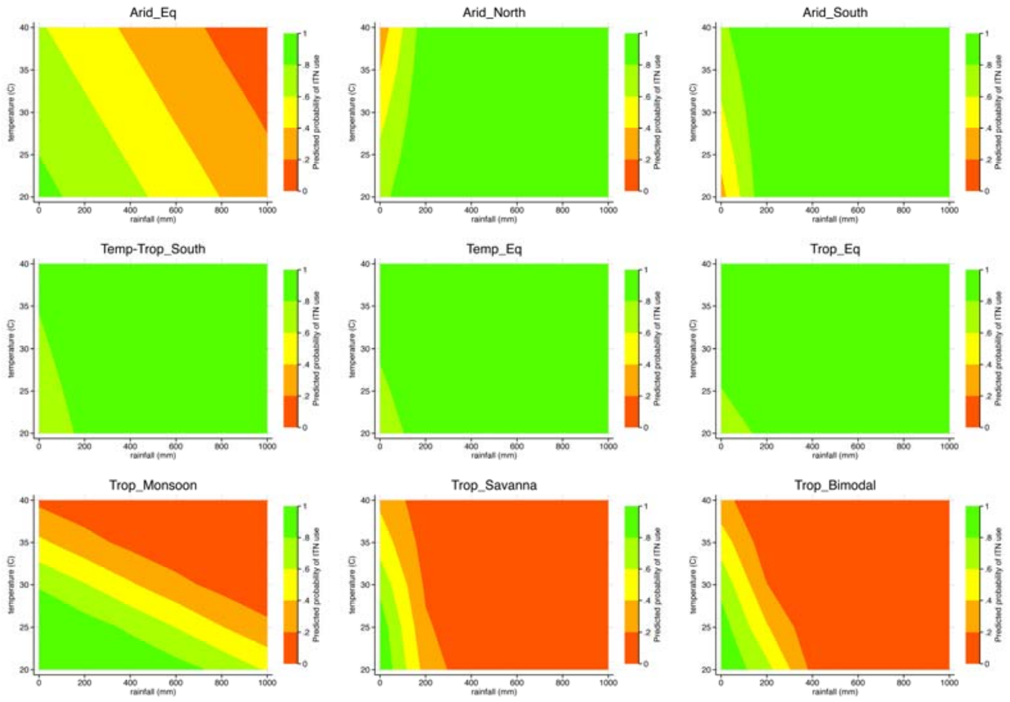

twoway contour plots of margins for avg rain (mm) and temperature from each zone model - no month

D. Graphs of base regression model monthly odds ratios compared to multi-level model with cluster level random effects. Arid South multi-level model was too unstable to run and hence is missing.

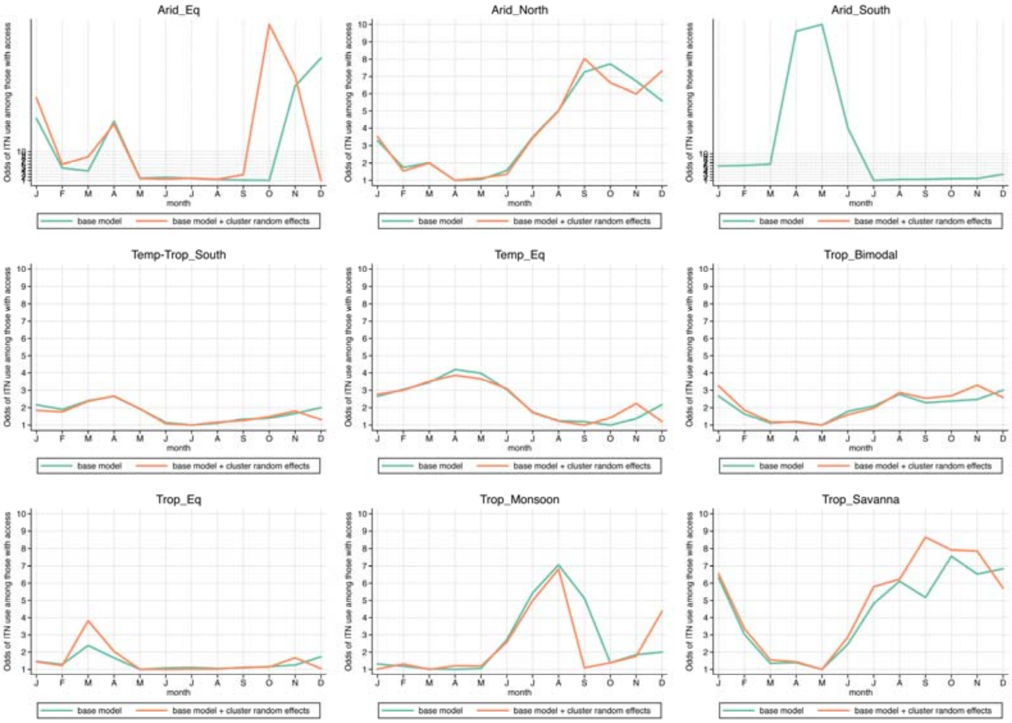

Supplement: Supplementary file 1 [file tpmd190249.SD1.pdf]
